# Obesity in Caucasian Seniors on the Rise: Is It Truly Harmful? Results of the PolSenior2 Study

**DOI:** 10.3390/nu14214621

**Published:** 2022-11-02

**Authors:** Monika Puzianowska-Kuznicka, Alina Kurylowicz, Lukasz Wierucki, Aleksander Jerzy Owczarek, Kacper Jagiello, Malgorzata Mossakowska, Tomasz Zdrojewski, Jerzy Chudek

**Affiliations:** 1 Department of Human Epigenetics, Mossakowski Medical Research Institute, 02-106 Warsaw, Poland; 2 Department of Geriatrics and Gerontology, Medical Centre of Postgraduate Education, 01-813 Warsaw, Poland; 3 Department of General Medicine and Geriatric Cardiology, Medical Centre of Postgraduate Education, 01-813 Warsaw, Poland; 4 Division of Preventive Medicine and Education, Medical University of Gdansk, 80-211 Gdansk, Poland; 5 Department of Pathophysiology, Medical University of Silesia, 40-752 Katowice, Poland; 6 Study on Ageing and Longevity, International Institute of Cell and Molecular Biology, 02-109 Warsaw, Poland; 7 Department of Internal Diseases and Oncological Chemotherapy, Medical University of Silesia, 40-027 Katowice, Poland

**Keywords:** abdominal obesity, Activities of Daily Living (ADL), aging, aging-associated diseases, obesity, Mini-Mental State Examination (MMSE), mortality, obesity paradox, PolSenior, PolSenior2

## Abstract

Obesity is associated with an increased risk of morbidity and mortality; however, data suggest that in old age, obesity is not detrimental. The study’s objective was to verify whether obesity frequency still increases in Polish Caucasian seniors and to verify the “obesity paradox”. Five thousand and fifty-seven community-dwelling individuals aged ≥ 65 years completed a detailed medical questionnaire, underwent measurements of the body mass index (BMI) and the waist circumference (WC), and an evaluation of physical and cognitive performances. Over a decade, general obesity increased by 2.1%, mostly due to a 3.9% increase in men. Abdominal obesity increased by 1.0%, mainly due to males, in whom it increased by 3.9%. Obesity increased the risk of several aging-related diseases, but this effect was less pronounced in the oldest-old. Obesity did not adversely affect the physical and cognitive functioning or mortality. Through a multivariable analysis, the BMI and WC remained the independent predictors of the Katz Activities of Daily Living score (*p* < 0.001 and *p* < 0.05, respectively) and Mini-Mental State Examination score (both *p* < 0.001). The Kaplan–Meier survival curves revealed that overweight and obesity classes 1 and 2 were associated with the lowest mortality. Through a multivariable analysis, overweight, class 1 obesity, and abdominal obesity remained the independent predictors of a decreased mortality (all *p* < 0.001). In conclusion, we found that overweight and obesity are not detrimental in seniors, including the oldest-old. We suggest that the anthropometric values defining obesity should be modified for age-advanced people.

## 1. Introduction

Populations in developed countries face an unprecedented rise in the number of age-advanced people. According to Eurostat, in the European Union (EU) member states, the number of people aged 65 and over will increase from 90.5 million in 2019 to 129.8 million in 2050, even though the total population will remain relatively stable at approximately 410–440 million. Moreover, the fastest growing is the population of people aged 85 and over; it is predicted that this will more than double by 2050. The EU population of centenarians will increase from ninety-six thousand to almost half a million [1]. Similar trends are observed in other developed countries outside of Europe [2,3]. However, population aging also threatens developing countries, which will inevitably face the consequences slightly later [4].

A longer life without an extension of its healthy phase will place pressure on health care and social care systems because aging is a crucial risk factor for the occurrence of aging-related diseases and disability [5]. Since older women outnumber older men, the existence of biological, lifestyle, or socioeconomic factors affecting aging in a gender-dependent manner is suggested [1]. It is, therefore, of fundamental importance to identify the factors influencing the course of aging. Notably, some of these factors may be modifiable.

Another worldwide public health threat is obesity, affecting all age groups [6,7]. The rate of obesity continues to rise: in 2010, 11% of the world’s population was obese, while the predictions are that 18% will be obese by 2030 [8]. The Americas, the eastern Mediterranean region, and Europe have the highest percentage of obese individuals, while the highest absolute numbers of obese men and women live in the USA, China, and India [8]. Being itself a complex and multifactorial disease, obesity significantly increases the risk of microinflammation and predisposes to the development of type 2 diabetes (DM), cardiovascular disease (CVD), non-alcoholic fatty liver disease, some cancers, osteoarthritis, sleep apnea, and premature death [9,10,11,12,13,14]. Obesity increases the risk of a severe course of infection, including COVID-19, and mortality associated with this disease [15]. It also negatively affects mental health and well-being and has an adverse socioeconomic impact [16]. However, a significant number of observations indicate that in age-advanced individuals, obesity has a protective rather than harmful influence and is associated with a better physical and cognitive performance, a decreased risk for the development and milder courses of some diseases, as well as a decreased risk of mortality, a phenomenon known as the “obesity paradox” [17,18,19,20]. We also showed that in community-dwelling seniors aged 65 years or older, a higher body mass index (BMI), waist circumference (WC), and arm circumference were associated with higher Katz Activities of Daily Living (ADL) scores and Mini-Mental State Examination (MMSE) scores and, except for class 3 obesity, with a decreased mortality rate [21]. The existence of the “obesity paradox” is not universally accepted, and various factors are indicated as being responsible for a positive association of a higher body mass with a better performance and survival rate in older individuals [22,23,24,25]. 

As obesity frequency increases in the whole adult population worldwide, we, therefore, by comparing the prevalence of obesity over a decade, aimed at verifying whether this trend also applies to age-advanced people, especially the oldest age group. Another aim was to check whether the “obesity paradox” can still be observed in an independent group of other community-dwelling Caucasian seniors. 

## 2. Materials and Methods

### 2.1. Study Design

The current study was conducted as a part of the PolSenior2 project, a cross-sectional study of a representative sample of Polish Caucasians, aged 60 years and over, designed to examine various aspects of aging, implemented between 2018 and 2019. PolSenior2 involved a representative sample of 5987 Polish Caucasians aged ≥ 60.0 years. The study subjects were divided into 5-year age cohorts: 60.0–64.9, 65.0–69.9, 70.0–74.9, 75.0–79.9, 80.0–84.9, 85.0–89.9, and ≥90.0 years. The cohorts were similar in number and consisted of similar numbers of women and men. The details of the recruitment process and study design have been published elsewhere. In short, the recruitment process for this study was designed to obtain a representative sample of Polish senior Caucasians. Thus, a three-stage stratified and proportional random sampling was performed: 1. administrative units, 2. random selection of villages in rural municipalities and cities in urban municipalities, and 3. study participants were drawn, based on their PESEL (Universal Electronic System for Registration of the Population) number. Among individuals with whom contact was possible, the response rate was 52% [26].

The study participants were visited three times by a trained medical person. During the visits, detailed medical and socioeconomic questionnaires were completed, blood and urine samples were taken, elements of medical examinations and basic anthropometric measurements were performed, and various tests evaluating the physical and cognitive performance were conducted. The mortality data were obtained from the population register of Poland. 

In the current study, the data regarding 5057 participants (2564 women, 2493 men) aged ≥ 65.0 years for whom the necessary information was provided, were analyzed (Supplementary Table S1). Sixty-five years was set as the threshold age because, according to the World Health Organization, in developed countries, older age is commonly defined as surpassing 65 years. In addition, this age limit allowed for a comparison of PolSenior (the first project designed to examine medical, psychological, and socioeconomic aspects of aging in Polish Caucasians) [27] and PolSenior2 results. The calculations of the BMI and the measurement of the WC were undertaken in 4905 and 4884 individuals, respectively. Study participants were categorized according to the BMI as: underweight (BMI < 18.5 kg/m^2^), normal weight (18.5–24.9 kg/m^2^), overweight (25.0–29.9 kg/m^2^), class 1 obesity (30.0–34.9 kg/m^2^), class 2 obesity (35.0–39.9 kg/m^2^), or class 3 obesity (≥40.0 kg/m^2^). A WC ≥ 80.0 cm in women and ≥94.0 cm in men was considered to be abdominal obesity, according to the International Diabetes Federation (IDF), while it was ≥88.0 cm in women and ≥102.0 cm in men, according to the National Cholesterol Education Program Adult Treatment Panel III (NCEP-ATP III) criteria [28,29].

Aging-associated diseases and adverse conditions, included DM, CVD (e.g., atrial fibrillation, coronary artery disease, myocardial infarction, heart failure, stroke), cancer (skin cancers other than melanoma, such as squamous and basal cell carcinomas, were not taken into account because they are primarily dependent on environmental factors), chronic obstructive pulmonary disease (COPD), chronic kidney disease (CKD; estimated glomerular filtration rate < 60 mL/min), the MMSE score < 24 points, and the ADL score < 5 points. The survival data were obtained from the population register of Poland, approximately 3.3 years after recruitment into the study. At the time of access to the register, 901 individuals had died. The anonymity and confidentiality of the personal data were preserved at all stages [26]. 

### 2.2. Activities of Daily Living and the Mini-Mental State Examination

Physical performance was assessed using an ADL scale in 4903 participants [30]. According to the score, they were stratified into independent, partially dependent, and totally dependent groups (ADL scores 5–6, 3–4, and 0–2 points, respectively). Following a cursory assessment of the vision and hearing, the cognitive function of 4871 suitable participants was assessed using the MMSE scale [31]. The study participants were divided into normal cognition, minimal cognitive impairment, mild cognitive impairment, moderate cognitive impairment, and severe cognitive impairment groups (MMSE scores 28–30, 24–27, 20–23, 10–19, and < 10 points, respectively) [32,33]. 

### 2.3. Statistical Analysis 

Statistical analyses were performed using SAS 9.4 TS Level 1M5 (SAS Institute, Cary, NC, USA) and R version 3.6.3 R (R Foundation for Statistical Computing, Vienna, Austria). Due to the skewed distribution of values for the BMI and the WC, variables were calculated as the median (first quartile—third quartile). The significance of the relationship between the analyzed factors was tested using a nonparametric Kruskal–Wallis Test. We performed a chi-squared test for the trend in proportions for the categorical data and a Mann Kendall trend test for the quantitative data, respectively, to check whether the data had a significant trend. We used Spearman’s rank correlation coefficient (rho) as a measure of the correlation between the parameters and a multivariate linear regression to model the relationship between two or more variables. Univariable and multivariable logistic regression models were created to determine the association between binary dependent variables and covariates. The proportions were compared with a chi-squared test and a chi-squared test for the trends in age groups. A Kaplan–Meier plot was used to present the survival curves, which were compared using a log-rank test. A Cox proportional hazards model was used for the univariable and multivariable survival analyses, with age as one of the covariates. A proportional-hazards assumption was tested using Schoenfeld residuals. For all statistical analyses, the level of significance was set at 0.05.

The statistical analysis of PolSenior and PolSenior2 studies took into account a complex survey sampling, and post-stratification was used to weigh the sample in relation to the structure of the Polish population in 2019.

## 3. Results

### 3.1. Frequency of General Obesity and Abdominal Obesity

The median BMI in consecutive age groups is shown in Table 1. The general obesity frequency assessed during the implementation of the PolSenior2 project, based on a BMI value ≥ 30.0 kg/m^2^, affected 39.9% of Polish Caucasians aged ≥ 65.0 years, which is a value slightly greater than the 37.8% found about ten years earlier during the implementation of the PolSenior project [21]. This difference was, however, not significant (Supplementary Table S2). This increase was mostly due to increased obesity in men: 31.5% vs. 35.4% in PolSenior and PolSenior2, respectively (not significant), and to a much lesser extent in women: 42.0% vs. 42.9%, respectively (not significant). The increase in obesity between the two PolSenior cohorts was observed in all age groups except 65.0–69.9 and ≥90.0 years old women. However, a level of significance was reached only in 75.0–79.9 and 85.0–89.9 years old men (*p* < 0.01 and *p* < 0.05, respectively) and 85.0–89.9 years old women (*p* < 0.05) (Table 2). The obesity frequency decreased with increasing age in both sexes (both *p* < 0.001).

The mean waist circumference in the consecutive age groups is presented in Table 1. It significantly changed in an age group-dependent manner in both men and women. Over a decade, it also rose in 75.0–79.9 and 85.0–89.9 year old men. Abdominal obesity, based on IDF WC values ≥ 80.0 cm in women and ≥94.0 cm in men, occurred in 87.1% of the studied population. A comparison of the PolSenior and PolSenior2 results revealed a minimal increase from 86.1% observed a decade earlier (Supplementary Table S3). This increase mainly occurred in men: 77.1% vs. 81.0% in PolSenior and PolSenior2 participants, respectively, while in women, abdominal obesity decreased: 92.1% vs. 91.1%. Notably, adopting the NCEP-ATP III reference values of WC ≥ 88.0 cm in women and ≥102.0 cm in men revealed a minimal increase in severe abdominal obesity from 68.6% to 69.3%, again due to the male sex: 53.2% vs. 56.2% in PolSenior and PolSenior2 participants, respectively, while in women, it decreased: 78.8% vs. 77.9%, respectively. None of these differences reached a level of significance.

The frequency of abdominal obesity changed with progressing age in both sexes (Table 3). An analysis of the 5-year age cohorts revealed a significant increase in severe abdominal obesity of ≥102.0 cm in older senior men aged ≥ 90.0 years (*p* < 0.05) between the PolSenior and PolSenior2 participants (Table 3). However, in all subsequent analyses, the IDF criteria were taken into account.

### 3.2. Association of the Body Measurements with the Physical and Cognitive Performance 

We next checked whether obesity is associated with the physical and cognitive performance of PolSenior2 participants (Table 4 and Table 5). 

The evaluation of the physical performance showed that, in the whole senior population, the correlation of the BMI with an ADL score, although significant, should be considered either extremely weak or negligible (*r_s_* = 0.06, *p* < 0.001). Sex stratification showed that the BMI very weakly correlated with the ADL in men (*r_s_* = 0.12, *p* < 0.001), while there was no correlation in women. The correlation of the WC with an ADL score was also extremely weak in both sexes together and in men (*r_s_* = 0.04, *p* < 0.01; *r_s_* = 0.09, *p* < 0.001, respectively), while in women no correlation existed. Next, we performed the multivariable analysis with age, sex, and smoking status as co-variates selected based on the literature data [34,35,36]. The analysis revealed that both body measurements remained independent predictors of an ADL score (BMI: *p* < 0.001, WC: *p* < 0.05).

Evaluation of the cognitive performance showed that the BMI very weakly correlated with a MMSE score in the whole examined population and in women and men separately (all *r_s_* = 0.18, *p* < 0.001). The WC did not correlate with a MMSE score. Through the multivariable analysis, which included age, sex, and smoking status as co-variates, both body measurements remained independent predictors of a MMSE score (all *p* < 0.001).

The additional stratification into younger and oldest-old seniors revealed that associations with a MMSE score, although still extremely weak or very weak, were stronger in the oldest age group (Table 6).

### 3.3. Association of the Body Measurements with Morbidity

General obesity was associated with a higher frequency of several aging-related conditions: diabetes, CVD, and CKD (*p* < 0.001, *p* < 0.01 and *p* < 0.05, respectively), and a lower frequency of the ADL score < 5 points indicating dependency (*p* < 0.01) or the MMSE score < 24 points indicating cognitive impairment (*p* < 0.01). No correlation existed with cancers and COPD. In women, obesity was associated with diabetes, CVD, and a lower frequency of the MMSE score < 24 points (*p* < 0.001, *p* < 0.001, and *p* < 0.05, respectively) but not with cancer, COPD, CKD, or the ADL score < 5 points. In men, a BMI ≥ 30 kg/m^2^ was associated with a higher frequency of diabetes, CVD, CKD, and a lower frequency of the ADL score < 5 points (*p* < 0.001, *p* < 0.001, *p* < 0.05, and *p* < 0.01, respectively). There was no correlation with cancer, COPD, or the MMSE score < 24. The stratification into the younger and oldest-old seniors revealed that, while obesity in the younger study participants correlated with a higher frequency of diabetes, CVD, CKD, and the MMSE score < 24 points (*p* < 0.001, *p* < 0.01, *p* < 0.001, and *p* < 0.05, respectively), in the ≥ 85 years old group, it correlated only with diabetes and CVD (*p* < 0.01 and *p* < 0.05, respectively). Notably, obesity in younger women correlated with diabetes and CKD (*p* < 0.001 and *p* < 0.01, respectively), while in the oldest-old women, only an association with CVD was found (*p* < 0.05). In younger senior men, general obesity correlated with a higher frequency of diabetes, CVD, CKD, and a lower frequency of low ADL scores (*p* < 0.001, *p* < 0.001, *p* < 0.05, and *p* < 0.01, respectively), but in oldest-old men, a correlation was only observed with diabetes and CKD (both *p* < 0.01).

Abdominal obesity was associated with a higher frequency of diabetes and a lower frequency of a poorer physical performance (*p* < 0.001 and *p* < 0.01, respectively). Abdominal obesity was not associated with CVD, cancer, COPD, CKD, or the MMSE score < 24 points. When both sexes were analyzed separately, in women, abdominal obesity was associated only with diabetes and a lower frequency of the ADL score < 5 points (*p* < 0.001 and *p* < 0.05, respectively), while in men, it was associated with diabetes, CVD, and a lower frequency of the ADL score < 5 points (*p* < 0.001, *p* < 0.05, and *p* < 0.05, respectively) but not with other analyzed aging-related conditions. The stratification into the younger and the oldest-old seniors showed that in the former, abdominal obesity correlated only with a higher frequency of diabetes (*p* < 0.001), while in the oldest-old group, a correlation with diabetes and CKD was noted (*p* < 0.05 and *p* < 0.001, respectively). Further stratification into the sexes showed that in younger women, abdominal obesity correlated only with the occurrence of diabetes (both *p* < 0.001), and in oldest-old women, this correlated only with CKD (*p* < 0.05). In younger senior men, abdominal obesity correlated with a higher frequency of diabetes, CVD, and a lower frequency of low ADL scores (*p* < 0.001, *p* < 0.05, and *p* < 0.05, respectively), while in the oldest men, a correlation was noted with diabetes and CKD (*p* < 0.05 and *p* < 0.01, respectively). 

### 3.4. Association of the Body Measurements with Mortality

To verify the effect of simple body measurements on the mortality in seniors, we used the Kaplan–Meier curves to evaluate survival. For this purpose, we stratified our study group into younger and oldest-old seniors. 

**Figure 1:**
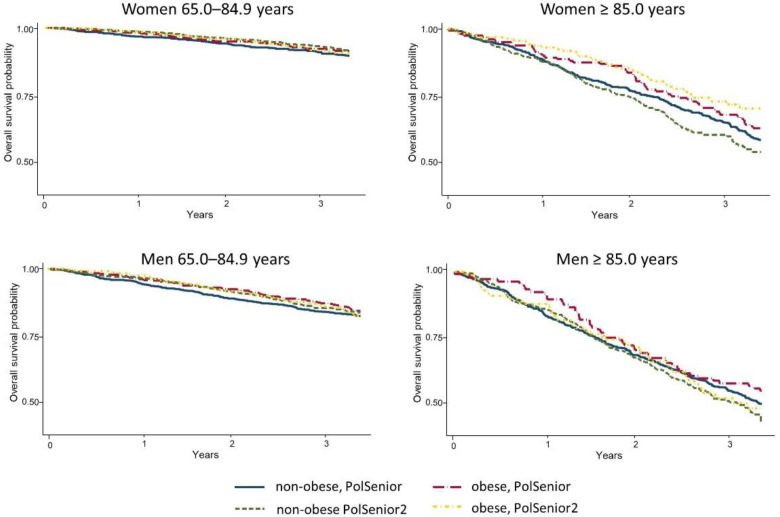
Kaplan–Meier survival curves comparing the survival probability in two cohorts of Polish Caucasian seniors (PolSenior and PolSenior2), recruited approximately ten years apart. Study participants were stratified into younger and oldest-old seniors (65.0–84.9 and ≥85.0 years, respectively), and non-obese and obese, according to the body mass index (BMI < 30.0 kg/m^2^ and ≥30.0 kg/m^2^, respectively).

**Figure 2:**
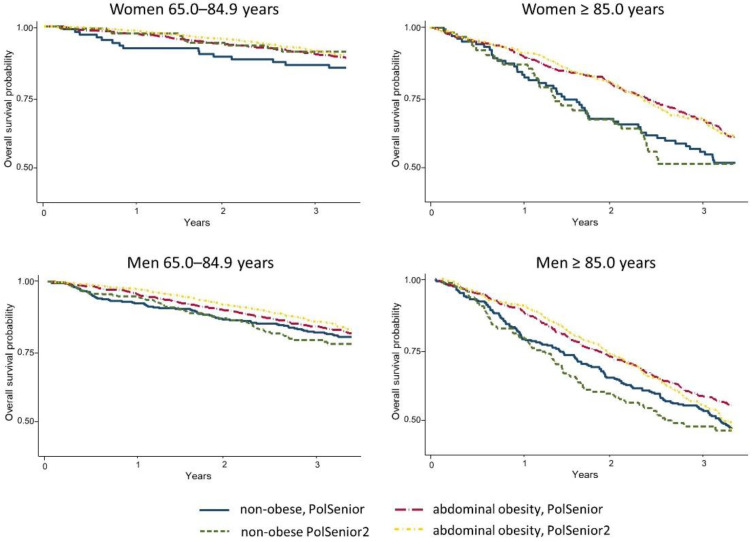
Kaplan–Meier survival curves comparing the survival probability in two cohorts of Polish Caucasian seniors (PolSenior and PolSenior2), recruited approximately ten years apart. Study participants were stratified into younger and oldest-old seniors (65.0–84.9 and ≥85.0 years, respectively), and without or with abdominal obesity, according to the International Diabetes Federation criteria (waist circumference [WC] < 80.0 cm in women and < 94.0 cm in men vs. WC ≥ 80.0 cm in women and ≥94.0 cm in men, respectively).

First, we checked whether survival in seniors associated with general or abdominal obesity, changed over the ten years. The Kaplan–Meier survival curves revealed that survival of PolSenior and PolSenior2 participants divided into non-obese or obese, based on the BMI values, was similar in the younger and oldest-old senior men, as well as in younger and oldest-old senior women (Figure 1, Supplementary Table S4). No differences in the survival curves were noticed between PolSenior and PolSenior2 participants of both sexes, stratified into younger and oldest-old seniors, and those with or without abdominal obesity (Figure 2, Supplementary Table S5).

**Figure 3:**
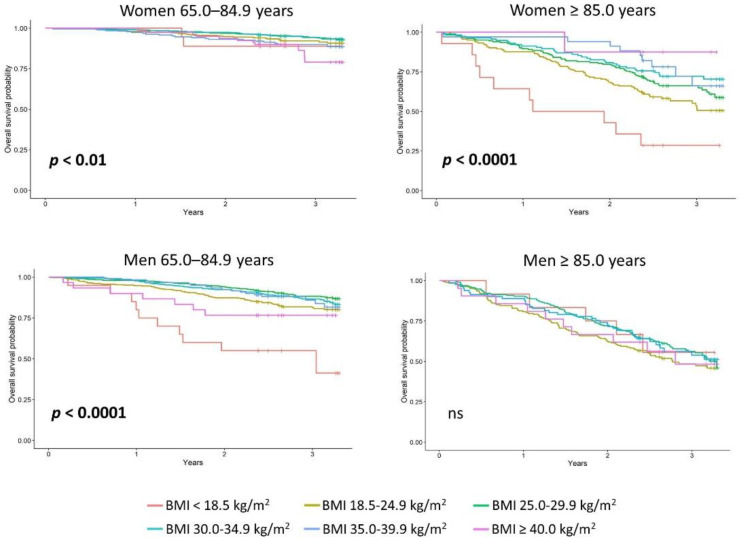
Kaplan–Meier survival curves in the participants of the PolSenior2 project, stratified into younger and oldest-old seniors (65.0–84.9 and ≥85.0 years, respectively) and according to the body mass index (BMI) value as underweight (BMI < 18.5 kg/m^2^), normal weight (18.5–24.9 kg/m^2^), overweight (25.0–29.9 kg/m^2^), class 1 obesity (30.0–34.9 kg/m^2^), class 2 obesity (35.0–39.9 kg/m^2^), and class 3 obesity (≥40.0 kg/m^2^).

**Figure 4:**
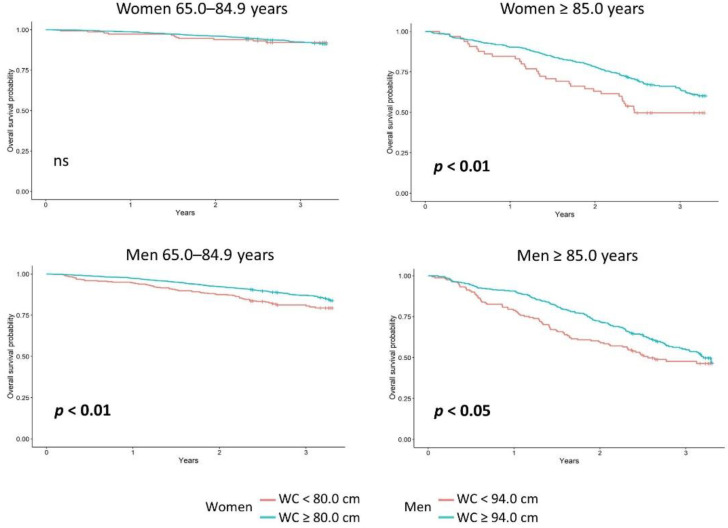
Kaplan–Meier survival curves in the participants of the PolSenior2 project, stratified into the younger and oldest-old seniors (65.0–84.9 and ≥85.0 years, respectively), without and with abdominal obesity, according to the International Diabetes Federation criteria (waist circumference [WC] < 80.0 cm in women and < 94.0 cm in men vs. WC ≥ 80.0 cm in women and ≥94.0 cm in men, respectively).

In the PolSenior 2 cohort, the Kaplan–Meier survival estimate was better for the oldest-old obese women than those without general obesity (*p* < 0.01, Figure 1). In the younger senior women, class 3 obesity was associated with the lowest probability of survival, while being overweight and class 1 obesity was associated with the highest probability of survival (*p* < 0.01). In the oldest-old women, however, underweight was associated with the lowest, while class 3, followed by class 1 and 2 obesity, were associated with the highest probability of survival (*p* < 0.0001). In younger senior men, underweight was associated with the lowest, while overweight and class 1 and 2 obesity, were associated with the highest probability of survival. In the oldest-old men, the BMI did not correlate with survival (Figure 3, Supplementary Table S6). Abdominal obesity was not associated with survival in the younger senior women. In contrast, in women aged 85 years or more, it was associated with an improved survival (*p* < 0.01). This type of obesity was also associated with the improved survival in younger and oldest-old senior men (*p* < 0.01 and *p* < 0.05, respectively, Figure 4, Supplementary Table S7).

The multivariable analysis of the age, sex, the number of aging-related diseases and conditions, smoking, and BMI or WC was performed. Notably, when compared to the BMI within the normal range (18.5–24.9 kg/m^2^), overweight and class 1 obesity decreased the risk of death (hazard ratio [HR] 0.69, 95% CI 0.58–0.81, *p* < 0.001 and HR 0.66, 95% CI 0.54–0.81, *p* < 0.001), obesity class 2 and 3 did not modify the risk of death, but being underweight increased this risk (HR 2.48, 95% CI 1.65–3.71, *p* < 0.001). In the analysis with the WC, abdominal obesity was associated with a decreased risk of death (HR 0.64, 95% CI 0.54–0.76, *p* < 0.001).

## 4. Discussion

While analyzing the two independent PolSenior and PolSenior2 groups of Polish Caucasians aged 65.0 years and over, collected ten years apart, we found that the frequency of obesity in this age group increased only slightly by 2.1%. Notably, even though they were still less obese than women, older men gained weight much faster as general obesity affected 3.9% more of them during the implementation of PolSenior2, while in older women, the increase was only 0.9%. The rise in abdominal obesity was 1.0% and 0.7%, according to the IDF and NCEP-ATP III criteria, respectively. Again, still more women than men had abdominal obesity, but the increase in the frequency of this ailment in men was 3.9% and 3.0% (IDF and NCEP-ATP III criteria, respectively), while in women, it actually decreased. The increase in obesity is observed worldwide; however, significant differences exist between countries not only in the percentage of obese people but also which sex is more obese and which gains weight faster [6,37,38,39,40]. Our data indicate that Polish Caucasian seniors, representing the senior population of the “new high-income countries”, are strongly affected by general and abdominal obesity and that preventive measures should take into account the needs of men. In addition, a new trend may have appeared in younger senior women aged 65.0–69.9 years in whom both the BMI and WC values, as well as the frequency of general and abdominal obesity, slightly decreased. Such a phenomenon was not observed in younger senior men. This trend is consistent with the results of the analysis in 18 European countries and the USA, performed between 1975 and 2016, which revealed a most prominent slowing of the increasing trend in the obesity frequency in young women [39]. Several potential explanations exist for the decrease in obesity observed in younger senior women. First, current beauty standards in developed and aspiring countries promote slimness which, together with the imperative of looking young, encourages dieting to achieve a slim body size and shape [41,42]. Second, women seem to pay more attention to health issues and are more aware of the negative consequences of obesity than men [43,44,45]. Third, with increasing wealth (defined as gross domestic product), the BMI of women decreases, possibly reflecting better access to health information and services, a higher level of education, as well as the attainment of healthy food [46]. 

In the present analysis, we confirmed our previous observation [21], that being underweight is associated with an increased risk of death. Because underweight is a common consequence of cancer, we analyzed the clinical data and found that only 3.1% of underweight individuals currently had cancer. Such a diagnosis had no significant influence on mortality in underweight study participants, compared with other study subjects. 

Moreover, general obesity and abdominal obesity were associated with several aging-related conditions, confirming the observations of other authors [47,48,49]. However, a higher total body mass and abdominal adipose tissue mass seemed not to pose a threat or even were positively associated with physical and cognitive functioning or survival, confirming our observation of the “obesity paradox” made during the implementation of the PolSenior project [21]. This is consistent with observations by other authors [17,18,19,20]. Therefore, in contrast to young and middle-aged populations, overweight and class 1 (and, possibly, class 2) obesity, in age-advanced individuals, are probably not detrimental and preventive actions should be aimed mostly at preventing morbid obesity as well as malnutrition. Notably, far fewer correlations existed between obesity and aging-related diseases in the oldest-old age group than in the younger seniors, even though we observed an increase in the frequency of the majority of aging-related diseases in oldest-old study participants (Supplementary Table S8). 

Our observations support the hypothesis that, starting from the ninth to the tenth decade of life, environmental factors become less important, and genetic background gains importance in governing the aging phenotype and longevity [50,51]. Another interesting observation is that the survival of the oldest obese women, especially those aged ≥ 90.0 years, significantly improved over a decade. This can result from a significantly increased WC, indicating the accumulation of abdominal adipose tissue, which is an important site for the peripheral production of estrogens (also discussed below) and is the most important source of these protective hormones in postmenopausal women. Another putative explanation for this phenomenon is that, regardless of the abovementioned increased frequency of these diseases in the oldest study participants, the management of obesity-related comorbidities significantly improved. Finally, the delayed onset of aging-related weight loss may have influenced this phenomenon. 

However, publications also suggest a negative impact of obesity on the functioning and survival rate of the elderly [52]. Among the plausible reasons for observing the “obesity paradox,” which, according to these authors, does not exist, were selection bias and methodological errors, such as omitting confounders, such as adipose tissue localization, smoking status, or accompanying diseases [22,53]. In our previous and current work, however, study participants were not preselected according to their health or smoking statuses. We also analyzed different anthropometric measurements being surrogates not only of general obesity, but also of abdominal obesity and peripheral muscle mass. Therefore, our data seem to support the “obesity paradox.”

There are at least two putative explanations why general and abdominal obesity might not be detrimental in seniors, based on endogenous reasons. First, a growing body of evidence exists, suggesting that the function of adipose tissue is among the key factors affecting health. Its dysfunction is associated with the overproduction of pro-inflammatory cytokines and a disturbed balance between various adipokines [54,55,56]. Sustained increased levels of pro-inflammatory cytokines in age-advanced individuals result in so-called inflammaging, an important risk factor for the development of chronic aging-related diseases [57,58]. However, inflammaging does not occur in all aging people. Indeed, we previously noticed that in successfully aging individuals, including obese ones, the levels of interleukin 6 and C-reactive protein are significantly lower than in the normally aging counterparts [11]. Moreover, inflammaging can be accompanied by increased levels of anti-inflammatory cytokines (anti-inflammaging) [59,60]. In conclusion, the balance between pro- and anti-inflammatory cytokines and other factors produced by adipose tissue may be more important than the weight of body fat itself. Second, adipose tissue is an important site for the peripheral production of estrogens in both sexes [61,62,63], and their levels correlate with body adiposity; in postmenopausal women, they are highest in those who are obese [64]. Moreover, in overweight and obese individuals, adipose tissue is stored not only in the abdominal cavity and subcutaneously but also around and inside various organs. Therefore, in individuals with excess fat, higher levels of circulating estrogens and locally produced estrogens may have a stronger protective effect on numerous tissues than in slim individuals [65,66].

We cannot exclude the notion that the “obesity paradox” might be caused by external factors. A growing body of evidence supports the view that diet composition is among the most important factors affecting health also at an older age [67,68,69]. Multiple factors make older persons less likely to meet the recommended daily values of nutrients [70]. Therefore, even if not of the highest quality, excess food might supply the necessary amounts of vitamins, microelements, polyphenols, and other nutrients, ensuring the proper function of molecular pathways involved in the regulation of the aging phenotype, albeit at the cost of obesity. Next, excess adipose tissue is associated with an increased risk of aging-related diseases, such as type 2 diabetes and cardiovascular diseases. Overweight and obese patients experiencing these chronic diseases see their doctors regularly and more commonly take medicines than their normal-weight same-age counterparts. These medications, including metformin and statins, not only positively affect patients’ health, but also have anti-aging potential and, therefore, can extend life [71]. Indeed, obese younger and oldest-old study participants took these medications more often than their non-obese counterparts (Supplementary Table S9). The contribution of such treatments to the “obesity paradox” is also supported by our earlier observation, that in successfully aging individuals not suffering from any aging-related diseases and, therefore, usually taking limited amounts of medications or taking them only occasionally, the “obesity paradox” was not observed, as we did not see an association of survival with the BMI, WC or arm circumference [21]. Furthermore, evidence exists that in an aging population, the level of physical activity is more important than the adipose tissue mass [72,73] and that fitness might mediate the “obesity paradox” by affecting adipokines and pro-inflammatory cytokine levels [74].

Finally, when looking for mortality predictors associated with body weight in an aging population, it should be taken into account that longitudinal observational studies suggest that body weight and composition in middle age are more strongly associated with disability and mortality at an older age than the BMI at older ages [75,76,77]. In turn, data regarding the consequences of weight loss in the elderly are conflicting [78,79,80]. 

This study has several limitations. First, one needs to regard the representativeness of the studied group. Older age groups were overrepresented to recognize better the health and social status of the most senior citizens. Furthermore, the inclusion of similar numbers of women and men resulted in the overrepresentation of men, especially in the oldest age groups [26]. To deal with this problem, however, we applied a weighting adjustment method [81]. Finally, participation in the program was voluntary. Several initially enrolled people were not available, and approximately 48% of those who were eligible did not agree to participate, resulting in a response rate of 52%. This, in turn, might have led to some selection bias. The second limitation regards the methods used to evaluate obesity. For this purpose, we used very simple anthropometric measurements instead of techniques allowing for the accurate evaluation of adipose and muscle tissue mass, such as bioelectric impedance, dual-energy X-ray absorptiometry, or nuclear magnetic resonance. However, in a general practitioner’s everyday practice measuring the BMI and WC, or calf and arm circumference is cost-free, fast, easy, and can be repeated during subsequent visits, enabling the observation of body mass changes. In addition, multiple reports describe data based on the evaluation of these anthropometric measurements, and a similar design of our study allows for the comparison of results.

Our work has several strengths. First, studying two large groups of older individuals using the same tools, allowed for the identification of changes in the health status of a growing ≥65.0-year-old population. Second, we relied on the inclusion of a high number of oldest-old individuals, allowing for a reliable evaluation of their health status. Third, the assessment of the study participants was thorough, since we collected detailed medical histories, performed blood and urine tests, and evaluated their current health status and physical and cognitive performance, among other variables [26]. Therefore, we are confident that our results are reliable. 

## 5. Conclusions

We found that obesity in Caucasian seniors, especially men, is rising. However, overweight and obesity class 1 are not detrimental to this population. Even though they increase the risk of aging-related diseases, they are associated with a better functional and cognitive performance and a lower mortality than normal weight or class 3 obesity and, especially, underweight. We also observed the increased survival of the oldest-old obese women, a phenomenon awaiting an explanation. If the “obesity paradox” truly exists, understanding its molecular and cellular basis would be of great importance for the creation of new methods to improve function and extend the life of the elderly. In addition, it seems advisable to consider whether the currently used values of the BMI, WC, and other parameters defining the limits of obesity should be modified for age-advanced people. 

## Figures and Tables

**Table 1:** BMI and waist circumference (WC) in consecutive age cohorts during the implementation of the PolSenior (2008–2009) and PolSenior2 (2018–2019) projects.

		BMI (kg/m^2^)		WC (cm)	
	Age (Years)	PolSenior	PolSenior2	PolSenior	PolSenior2
Women	All	28.9 (25.6–32.8)	29.0 (25.3–32.6)	98.0 (89.5–106.0)	98.0 (89.0–107.0)
	65.0–69.9	30.2 (26.6–33.4)	29.1 (25.3–32.7) *	99.0 (90.0–107.5)	97.0 (88.0–107.0)
	70.0–74.9	29.2 (25.5–33.6)	30.0 (26.2–33.9)	98.0 (90.0–106.0)	98.5 (89.5–107.0)
	75.0–79.9	28.4 (25.5–32.4)	28.7 (25.2–32.7)	97.0 (89.0–104.0)	97.0 (90.0–106.0)
	80.0–84.9	28.2 (25.6–32.0)	28.9 (25.6–32.0)	97.0 (90.0–107.0)	98.5 (90.0–107.1)
	85.0–89.9	26.5 (24.2–30.3)	28.5 (24.2-32.0)	95.0 (86.0-103.0)	98.0 (88.0-107.0)
	≥ 90.0	25.1 (21.4–30.3)	26.8 (22.8–30.3)	92.0 (83.0–102.0)	95.0 (84.5-104.0)
	*p* ⇓	<0.001	<0.001	0.12	<0.001
Men	All	27.8 (25.2–30.9)	28.0 (25.3–31.3)	103.0 (94.5–110.0)	103.0 (96.0–112.0)
	65.0–69.9	28.2 (26.0–32.2)	28.3 (26.1–31.8)	104.0 (96.5–111.0)	104.0 (97.0–112.0)
	70.0–74.9	28.3 (25.1–31.5)	28.0 (25.3–31.5)	102.0 (96.0–111.0)	103.2 (96.0–111.0)
	75.0–79.9	27.5 (25.4–29.8)	28.4 (25.3–31.5)	103.0 (94.0–110.0)	104.0 (97.0–113.0) *
	80.0–84.9	27.4 (24.6–30.0)	27.4 (24.4–30.5)	101.0 (94.0–108.0)	102.0 (95.0–111.0)
	85.0–89.9	26.2 (23.7–28.9)	26.5 (23.7–29.7)	98.5 (90.0–105.0)	100.0 (92.0–109.0) *
	≥ 90.0	25.0 (22.8–28.1)	25.6 (23.1–28.6)	96.0 (88.0–104.0)	98.0 (90.0–107.0)
	*p* ⇓	<0.05	<0.001	0.10	<0.001

BMI: body mass index, median (Q_1_–Q_3_); WC: waist circumference, median (Q_1_–Q_3_); *p* ⇓ (Mann Kendall trend test): *p*-value for the age-related differences in the percentage of obese individuals in the consecutive age cohorts; *: *p* < 0.05.

**Table 2:** Obesity (BMI ≥ 30 kg/m^2^) frequency in women and men in the consecutive age groups during the implementation of the PolSenior (2008–2009) and PolSenior2 (2018–2019) projects.

	Obesity % (95% CI)			
	Men		Women	
Age (Years)	PolSenior	PolSenior2	PolSenior	PolSenior2
All	31.5 (26.6–36.3)	35.4 (32–38.8)	42.0 (39–45)	42.9 (40.0–45.8)
65.0–69.9	36.1 (25.9–46.4)	38.2 (32.6–43.8)	50.3 (43.4–57.2)	43.3 (37.2–49.4)
70.0–74.9	36.1 (27.4–44.7)	36.9 (30.6–43.3)	44.3 (35.7–52.8)	50.3 (44.0–56.7)
75.0–79.9	24.4 (18.3–30.4)	37.5 (32.3–42.6) **	38.9 (32.6–45.1)	40.5 (34.7–46.2)
80.0–84.9	25.1 (18.3–32.0)	28.4 (23.1–33.7)	35.1 (27.8–42.3)	42.0 (35.1–48.9)
85.0–89.9	15.9 (11.1–20.6)	24.5 (17.3–31.6) *	26.6 (20.7–32.6)	36.6 (28.9–44.3) *
≥ 90.0	11.5 (7.2–15.9)	15.0 (9.3–20.8)	26.7 (13.4–40.0)	25.9 (18.1–33.7)
*p* ⇓	<0.001	<0.001	<0.001	<0.001
**OR (±95% CI)**				
All	Ref.	1.37 ^#^ (1.21–1.56)	Ref.	1.09 (0.96–1.22)
65.0–69.9		1.27 (0.96–1.67)		0.82 (0.63–1.09)
70.0–74.9		1.07 (0.83–1.38)		1.01 (0.74–1.29)
75.0–79.9		1.60 ** (1.20–2.15)		1.02 (0.77–1.35)
80.0–84.9		1.07 (0.79–1.46)		1.08 (0.81–1.45)
85.0–89.9		1.30 (0.90–1.89)		1.29 (0.92–1.80)
≥ 90.0		1.81 * (1.09–3.01)		1.28 (0.85–1.94)

CI: confidence interval; *p* ⇓ (chi-squared test for the trend in proportions): *p*-value for age-related differences in the percentage of obese individuals in the consecutive age cohorts; *: *p* < 0.05; **: *p* < 0.01; ^#^: *p* < 0.001.

**Table 3:** Abdominal obesity frequency in the consecutive age groups during the implementation of the PolSenior (2008–2009) and PolSenior2 (2018–2019) projects.

	Abdominal Obesity NCEP-ATP III, M ≥ 102 cm, W ≥ 88 cm % (95% CI)				Abdominal Obesity IDF, M ≥ 94 cm, W ≥ 80 cm % (95% CI)			
	Men		Women		Men		Women	
Age (Years)	PolSenior	PolSenior2	PolSenior	PolSenior2	PolSenior	PolSenior2	PolSenior	PolSenior2
All	53.2 (48.4–58.0)	56.2 (52.5–59.9)	78.8 (76.2–81.4)	77.9 (75.6–80.2)	77.1 (73.7–80.5)	81.0 (78.1–83.9)	92.1 (90.5–93.6)	91.1 (89.5–92.8)
65.0–69.9	58.7 (49.2–68.2)	58.5 (52.3–64.7)	81.5 (77.6–85.5)	76.2 (71.6–80.9)	78.4 (72.0–84.9)	83.0 (78.6–87.5)	94.1 (91.4–96.8)	90.5 (86.9–94.2)
70.0–74.9	51.6 (43.2–60.1)	56.9 (51.1–62.8)	78.5 (73.8–83.2)	79.6 (75.2–84.0)	80.2 (74.8–85.7)	82.8 (78.0–87.6)	91.0 (87.1–95.0)	92.2 (88.6–95.7)
75.0–79.9	53.4 (45.6–61.2)	57.5 (52.2–62.8)	79.8 (74.6–85.1)	80.0 (75.1–85.0)	76.0 (70.5–81.5)	80.9 (75.8–86.1)	93.1 (90.1–96.1)	90.2 (85.7–94.6)
80.0–84.9	48.7 (41.9–55.4)	53.5 (46.6–60.5)	81.1 (75.6–86.6)	79.5 (74.0–85.0)	75.8 (69.9–81.8)	78.0 (72.8–83.1)	92.3 (89.0–95.6)	95.1 (92.1–98.0)
85.0–89.9	37.0 (30.9–43.0)	45.4 (37.8–53.1)	73.4 (65.1–81.7)	76.9 (70.3–83.4)	67.1 (61.2–73.1)	72.1 (64.1–80.0)	89.9 (85.9–93.8)	90.1 (85.5–94.6)
≥90.0	31.5 (25.7–37.3)	41.1 (33.2–49.1) *	57.2 (42.7–71.7)	71.4 (64.7–78.0)	55.8 (47.1–64.5)	64.9 (56.1–73.8)	81.1 (73.8–88.3)	83.2 (77.5–89.0)
*p* ⇓	<0.001	<0.001	<0.001	0.08	<0.001	<0.001	<0.001	0.067
	**OR (95% CI)**							
All	Ref.	1.43 ^#^ (1.28–1.60)	Ref.	1.09 (0.95–1.25)	Ref.	1.41 ^#^ (1.23–1.61)	Ref.	1.24 * (1.02–1.51)
65.0–69.9		1.24 (0.95–1.63)		0.89 (0.66–1.21)		1.57 * (1.11–2.20)		0.80 (0.51–1.27)
70.0–74.9		1.25 (0.97–1.61)		1.01 (0.73–1.38)		1.17 (0.85–1.62)		1.13 (0.69–1.87)
75.0–79.9		1.47 ** (1.13–1.93)		1.13 (0.79–1.60)		1.72 ** (1.24–2.37)		1.09 (0.67–1.79)
80.0–84.9		1.35 * (1.02–1.79)		0.96 (0.67–1.36)		1.06 (0.76–1.48)		1.58 (0.88–2.81)
85.0–89.9		1.46 * (1.07–1.99)		1.15 (0.80–1.66)		1.19 (0.85–1.68)		1.11 (0.67–1.85)
≥90.0		1.48 * (1.04–2.09)		1.31 (0.90–2.02)		1.38 (0.98–1.94)		1.54 (0.97–2.44)

CI: confidence interval; *p* ⇓ (chi-squared test for the trend in proportions): *p*-value for the age-related differences in the percentage of abdominal obesity in the consecutive age cohorts; *: *p* < 0.05; **: *p* < 0.01; ^#^: *p* < 0.001.

**Table 4:** Obesity and the Activities of Daily Living score in ≥65 years old individuals.

	BMI (kg/m^2^) *		WC (cm) *	
ADL (Points)	<30	≥30	W: <80, M: <94	W: ≥80, M: ≥94
5–6	59.5% (57.1–61.9)	40.5% (38.1–42.9)	12.5% (10.8–14.2)	87.5% (85.8–89.2)
3–4	63.8% (54.0–73.6)	36.2% (26.4–46.0)	12.9% (7.5–18.4)	87.1% (81.6–92.5)
0–2	77.9% (68.1–87.6)	22.1% (12.4–31.9)	27.2% (18.4–35.9)	72.8% (64.1–81.6)
**ADL (Points)**	BMI (kg/m^2^) **		WC (cm) **	
5–6	28.6 (25.4–32.2)		100.0 (91.5–109.0)	
3–4	27.9 (23.4–31.6)		102.0 (93.0–109.0)	
0–2	26.5 (23.4–29.0)		93.0 (83.0–105.5)	

*: % (95% CI); **: median (Q_1_, Q_3_); ADL: Activities of Daily Living; BMI: body mass index; WC: waist circumference; W: cut-off value for females; M: cut-off values for males.

**Table 5:** Obesity and the Mini-Mental State Evaluation score in ≥65 years old individuals.

	BMI (kg/m^2^) *		WC (cm) *	
MMSE (Points)	<30	≥30	W: <80, M: <94	W: ≥80, M: ≥94
28–30	59.6% (56.5–62.7)	40.4% (37.3–43.5)	12.5% (10.1–14.8)	87.5% (85.2–89.9)
24–27	57.6% (53.8–61.4)	42.4% (38.6–46.2)	12.8% (10.4–15.2)	87.2% (84.8–89.6)
20–23	62.3% (56.2–68.5)	37.7% (31.5–43.8)	10.5% (7.2–13.8)	89.5% (86.2–92.8)
10–19	75.0% (65.2–84.4)	25.0% (15.2–34.8)	20.4% (14.3–26.4)	79.6% (73.6–85.7)
<10	68.8% (54.6–82.9)	31.2% (17.1–45.4)	21.8% (11.7–32.0)	78.2% (68.0–88.3)
**MMSE (Points)**	BMI (kg/m^2^) **		WC (cm) **	
28–30	28.7 (25.6–32.0)		100.0 (92.0–109.0)	
24–27	28.8 (25.4–32.9)		101.0 (92.0–110.0)	
20–23	28.3 (25.2–31.3)		101.0 (91.0–110.0)	
10–19	26.5 (23.2–29.8)		96.0 (88.0–105.0)	
<10	27.5 (23.4–30.5)		95.5 (85.0–104.0)	

*: % (95% CI); **: median (Q_1_, Q_3_); MMSE: Mini-Mental State Evaluation; BMI: body mass index; WC: waist circumference; W: cut-off value for females; M: cut-off values for males.

**Table 6:** Spearman correlation between the ADL and MMSE scores and basic anthropometric measurements in younger and oldest-old seniors.

	ADL		MMSE	
	BMI	WC	BMI	WC
	Whole cohort *			
<85 years	*r_s_* = 0.01, *p* = 0.42	*r_s_* = 0.0, *p* = 0.91	*r_s_* = 0.02, *p* = 0.22	*r_s_* = −0.02, *p* = 0.18
≥85 years	*r_s_* = 0.02, *p* = 0.54	*r_s_* = 0.0, *p* = 0.91	*r_s_* = 0.11, *p* < 0.001	*r_s_* = 0.1, *p* < 0.001
	Women *			
<85 years	*r_s_* = −0.04, *p* = 0.10	*r_s_* = −0.06, *p* < 0.01	*r_s_* = 0.02, *p* = 0.29	*r_s_* = −0.03, *p* = 0.19
≥85 years	*r_s_* = 0.03, *p* = 0.45	*r_s_* = 0.01, *p* = 0.84	*r_s_* = 0.15, *p* < 0.001	*r_s_* = 0.09, *p* < 0.05
	Men *			
<85 years	*r_s_* = 0.08, *p* < 0.001	*r_s_* = 0.05, *p* < 0.05	*r_s_* = 0.02, *p* = 0.43	*r_s_* = −0.02, *p* = 0.47
≥85 years	*r_s_* = 0.05, *p* = 0.23	*r_s_* = 0.07, *p* = 0.12	*r_s_* = 0.13, *p* < 0.01	*r_s_* = 0.1, *p* < 0.05

*: Whole cohort < 85 years: n = 3927 and ≥85 years: n = 1130; Women < 85 years: n = 1998 and ≥85 years: n = 566; Men < 85 years: n = 1929 and ≥85 years: n = 564; BMI: body mass index; WC: waist circumference.

## Data Availability

Data are available from the corresponding author upon a reasonable request.

## Supplementary Materials

The following supporting information can be downloaded at: https://www.mdpi.com/article/10.3390/nu14214621/s1, Supplementary Table S1: The number of PolSenior2 study participants divided into 5-year age cohorts; Supplementary Table S2: General obesity (BMI ≥ 30 kg/m^2^) frequency in the consecutive age groups, estimated during the implementation of the PolSenior (2008–2009) and PolSenior2 (2018–2019) projects; Supplementary Table S3: Abdominal obesity frequency according to IDF criteria (M ≥ 94.0 cm, W ≥ 80.0 cm) in the consecutive age groups, estimated during the implementation of the PolSenior (2008–2009) and PolSenior2 (2018–2019) projects; Supplementary Table S4: The number of PolSenior (2008–2009) and PolSenior2 (2018–2019) study participants, stratified into younger and oldest-old seniors (65.0–84.9 and ≥85.0 years, respectively), and non-obese and obese, according to the body mass index (BMI < 30.0 kg/m^2^ and ≥30.0 kg/m^2^, respectively) whose survival probability was analyzed by the Kaplan–Meier survival curve method; Supplementary Table S5: The number of PolSenior (2008–2009) and PolSenior2 (2018–2019) study participants, stratified into younger and oldest-old seniors (65.0–84.9 and ≥85.0 years, respectively), and without or with abdominal obesity according to the International Diabetes Federation criteria (waist circumference [WC] < 80.0 cm in women and <94.0 cm in men vs. WC ≥ 80.0 cm in women and ≥94.0 cm in men, respectively), whose survival probability was analyzed using the Kaplan–Meier survival curve method; Supplementary Table S6: The number of PolSenior2 study participants, stratified into younger and oldest-old seniors (65.0–84.9 and ≥85.0 years, respectively), and according to the body mass index (BMI) value as underweight (BMI < 18.5 kg/m^2^), normal weight (18.5–24.9 kg/m^2^), overweight (25.0–29.9 kg/m^2^), class 1 obesity (30.0–34.9 kg/m^2^), class 2 obesity (35.0–39.9 kg/m^2^), and class 3 obesity (≥40.0 kg/m^2^), whose survival probability was analyzed using the Kaplan–Meier survival curve method; Supplementary Table S7: The number of PolSenior2 study participants stratified into younger and oldest-old seniors (65.0–84.9 and ≥85.0 years, respectively), and without or with abdominal obesity according to the International Diabetes Federation criteria (waist circumference [WC] < 80.0 cm in women and <94.0 cm in men vs. WC ≥ 80.0 cm in women and ≥94.0 cm in men, respectively), whose survival probability was analyzed using the Kaplan–Meier survival curve method; Supplementary Table S8: Frequency of aging-related diseases in the PolSenior2 study participants, stratified into younger and oldest-old seniors (65.0–84.9 and ≥85.0 years, respectively); Supplementary Table S9: Use of drugs with anti-aging potential in the PolSenior2 study participants, stratified into younger and oldest-old seniors (65.0–84.9 and ≥85.0 years, respectively).
