# Supplementary material for: Obesity in Caucasian Seniors on the Rise: Is It Truly Harmful? Results of the PolSenior2 Study
Source: Nutrients. 2022 Nov 2;14(21):4621. doi: 10.3390/nu14214621 (PMC9658066; doi:10.3390/nu14214621)
Supplement: Supplementary file 1 [file nutrients-14-04621-s001.zip › nutrients-1970384-supplementary.pdf]

Supplementary Table S1. The number of PolSenior2 study participants divided into 5-year age cohorts.

Age (years)	Women (n)	Men (n)	Whole cohort (n)
65.0-69.9	594	525	1119
70.0-74.9	512	540	1052
75.0-79.9	455	455	910
80.0-84.9	437	409	846
85.0-89.9	320	294	614
≥ 90.0	246	270	516
All	2564	2493	5057

Supplementary Table S2. General obesity (BMI ≥ 30.0 kg/m^2) frequency in the consecutive age groups, estimated during the implementation of the PolSenior (2008-2009) and PolSenior2 (2018-2019) projects.

	**General obesity % (95% CI)**	
**Age (years)**	**PolSenior**	**PolSenior2**
All	37.8 (35.5-40.1)	39.9 (37.5-42.4)
65.0-69.9	44.0 (38.4-49.7)	41.0 (37.0-45.1)
70.0-74.9	40.9 (35.6-46.1)	44.7 (40.1-49.3)
75.0-79.9	33.3 (28.5-38.1)	39.3 (34.9-43.7)
80.0-84.9	31.6 (27.1-36.2)	37.4 (32.6-42.1)
85.0-89.9	23.3 (18.4-28.1)	32.9 (27.9-38.0)**
≥ 90.0	22.7 (12.8-32.6)	23.3 (17.3-29.3)
p ⇓	< 0.001	< 0.001

CI: confidence interval. *p* ⇓ (chi-squared test for trend in proportions): *p*-value for age-related differences in the percentage of obese individuals in the consecutive age cohorts. ** $p < 0.01$

Supplementary Table S3. Abdominal obesity frequency according to IDF criteria (M ≥ 94.0 cm, W ≥ 80.0 cm) in the consecutive age groups, estimated during the implementation of the PolSenior (2008-2009) and PolSenior2 (2018-2019) projects.

	Abdominal obesity % (95% CI)	
**Age (years)**	**PolSenior**	**PolSenior2**
All	86.1 (84.3-87.9)	87.1 (85.5-88.8)
65.0-69.9	87.1 (83.9-90.3)	87.2 (84.2-90.2)
70.0-74.9	86.5 (83.4-89.6)	88.2 (85.3-91.1)
75.0-79.9	86.6 (83.8-89.5)	86.6 (82.6-90.7)
80.0-84.9	86.6 (83.6-89.5)	89.3 (87.0-91.6)
85.0-89.9	83.0 (80.0-85.9)	84.7 (80.4-88.9)
≥ 90.0	74.5 (68.7-80.2)	78.7 (74.0-83.4)
p ⇓	< 0.001	< 0.001

CI: confidence interval. *p* ⇓ (chi-squared test for trend in proportions): *p*-value for age-related differences in the percentage of abdominal obesity in the consecutive age cohorts.

Supplementary Table S4. The number of PolSenior (2008-2009) and PolSenior2 (2018-2019) study participants, stratified into younger and oldest-old seniors (65.0-84.9 and ≥ 85.0 years, respectively), and non-obese and obese according to body mass index (BMI < 30.0 kg/m^2 and ≥ 30.0 kg/m^2, respectively) whose survival probability was analyzed by the Kaplan-Meier survival curve method.

	**65.0-84.9 years**				**≥ 85 years**			
	**Non-obese (n)**		**Obese (n)**		**Non-obese (n)**		**Obese (n)**	
	PolSenior	PolSenior2	PolSenior	PolSenior2	PolSenior	PolSenior2	PolSenior	PolSenior2
Women	881	1117	686	842	476	360	159	157
Men	1155	1224	509	675	649	428	109	102

n: number of study participants

Supplementary Table S5. The number of PolSenior (2008-2009) and PolSenior2 (2018-2019) study participants stratified into younger and oldest-old seniors (65.0-84.9 and ≥ 85.0 years, respectively), and without or with abdominal obesity according to the International Diabetes Federation criteria (waist circumference [WC] < 80.0 cm in women and < 94.0 cm in men *vs.* WC ≥ 80.0 cm in women and ≥ 94.0 cm in men, respectively) whose survival probability was analyzed by the Kaplan-Meier survival curve method.

	**65.0-84.9 years**				**≥ 85 years**			
	**Non-obese (n)**		**Abdominal obesity (n)**		**Non-obese (n)**		**Abdominal obesity (n)**	
	PolSenior	PolSenior2	PolSenior	PolSenior2	PolSenior	PolSenior2	PolSenior	PolSenior2
Women	129	148	1453	1801	115	65	563	463
Men	396	343	1290	1529	279	161	499	374

n: number of study participants

Supplementary Table S6. The number of PolSenior2 study participants stratified into younger seniors and oldest-old (65.0-84.9 and ≥ 85.0 years, respectively), and according to body mass index (BMI) value as underweight (BMI < 18.5 kg/m^2), normal weight (18.5-24.9 kg/m^2), overweight (25.0-29.9 kg/m^2), class 1 obesity (30.0-34.9 kg/m^2), class 2 obesity (35.0-39.9 kg/m^2), and class 3 obesity (≥ 40.0 kg/m^2) whose survival probability was analyzed by the Kaplan-Meier survival curve method.

		**BMI (kg/m^2)**					
		**< 18.5**	**18.5-24.9**	**25.0-29.9**	**30.0-34.9**	**35.0-39.9**	**≥ 40.0**
Women (n)	65.0-84.9 years	18	383	716	575	188	79
	≥ 85.0 years	14	162	184	115	34	8
Men (n)	65.0-84.9 years	20	387	818	524	120	30
	≥ 85.0 years	12	187	229	81	21	0

n: number of study participants

Supplementary Table S7. The number of PolSenior2 study participants stratified into younger and oldest-old seniors (65.0-84.9 and ≥ 85.0 years, respectively), and without or with abdominal obesity according to the International Diabetes Federation criteria (waist circumference [WC] < 80.0 cm in women and < 94.0 cm in men *vs.* WC ≥ 80.0 cm in women and ≥ 94.0 cm in men, respectively), whose survival probability was analyzed by the Kaplan-Meier survival curve method.

	**65.0-84.9 years**		**≥ 85 years**	
	**Non-obese (n)**	**Abdominal obesity (n)**	**Non-obese (n)**	**Abdominal obesity (n)**
Women	148	1801	65	463
Men	343	1529	161	374

n: number of study participants

Supplementary Table S8. Frequency of aging-related diseases in the PolSenior2 study participants, stratified into younger and oldest-old seniors (65.0-84.9 and ≥ 85.0 years, respectively).

	Age (years)	n	Aging-related disease (%)				
			Diabetes	CVD	COPD	CKD	Cancer
Women	65.0-84.9	1998	44.3	34.8	3.3	20.0	9.2
	≥ 85.0	566	46.5	55.0	3.5	54.9	8.6
Men	65.0-84.9	1929	53.4	43.3	6.5	21.6	9.9
	≥ 85.0	564	46.8	59.8	6.6	54.3	10.7

Supplementary Table S9. Use of drugs with anti-aging potential in the PolSenior2 study participants stratified into younger and oldest-old seniors (65.0-84.9 and ≥ 85.0 years, respectively).

		Drugs with anti-aging potential (%)	
	**Age (years)**	**Metformin**	**Statins**
Non-obese	65.0-84.9	13.9	41.1
	≥ 85.0	10.7	35.3
Obese	65.0-84.9	27.3	48.1
	≥ 85.0	19.2	42.0
